# Serum levels of neutrophil Gelatinase associated Lipocalin (NGAL) predicts hemodialysis after coronary angiography in high risk patients with acute coronary syndrome

**DOI:** 10.1186/s12882-020-01799-5

**Published:** 2020-04-22

**Authors:** Luis F. Reyes, Diego F. Severiche-Bueno, Carlos A. Bustamante, Sixta Murillo, Nilam J. Soni, Marcela Poveda, Efraín Gomez, Ricardo Buitrago, Alejandro Rodriguez

**Affiliations:** 1 Universidad de La Sabana, Chía, Colombia; 2 Clínica Universidad de La Sabana, Chía, Colombia; 3 Division of Pulmonary & Critical Care Medicine and Division of General & Hospital Medicine, University of Texas Health San Antonio, San Antonio, USA; 4 Medicine Service, South Texas Veterans Health Care System, San Antonio, USA; 5 Clinica Shaio, Bogota, Colombia; 6 Critical Care Medicine, Hospital Universitari Joan XXIII, Tarragona, Spain

**Keywords:** Contrast-induced nephropathy, Hemodialysis, Biomarkers

## Abstract

**Background:**

Contrast-induced nephropathy (CIN) following a percutaneous coronary intervention (PCI) is the third most common cause of acute kidney injury (AKI) worldwide. Patients who require hemodialysis secondary to CIN have an elevated mortality rate as high as 55%. The current definition of CIN is based on an elevation of creatinine and decrease in urinary output. Creatinine typically increases 48 h after the contrast exposure, which delays the diagnosis and treatment of CIN. The neutrophil gelatinase associated lipocalin (NGAL) has emerged as a sensitive and specific biomarker of renal injury. Limited data exists about the effectiveness of NGAL to predict CIN in high-risk patients with acute coronary syndrome (ACS) that underwent PCI. The primary aim of this study was to determine the association of serum NGAL levels and the need for hemodialysis after PCI.

**Methods:**

This is a prospective, observational study. NGAL levels were measured using ELISA. Blood samples were obtained within the first 6 h of hospital admission, and 12 and 24 h after contrast exposure from angiography. The primary outcome was the requirement of hemodialysis. The non-parametric Mann-Whitney U test was used to test for differences in median serum levels of NGAL. A receiver operating characteristic (ROC) curve was developed to assess the accuracy of NGAL to predict the need for hemodialysis after PCI.

**Results:**

A total of 2875 were screened; however, 45 patients with ACS that underwent PCI were included. All patients were at high risk of developing CIN defined by Mehran score > 11 points. The median (IQR) serum concentration of NGAL was significantly higher in patients that required versus did not require hemodialysis (340 [83–384] vs. 169 [100–210], *p* = 0.01). Elevated serum levels of NGAL with a cut-off at 6 h post PCI of 281 mg/dL predicted the need for hemodialysis with an area under the curve of 0.86 (95% CI, 0.66–1.00).

**Conclusions:**

In patients with ACS undergoing PCI; and high risk of developing CIN, an elevated serum level of NGAL 6 h after contrast exposure predicts the development of acute kidney injury requiring hemodialysis.

## Background

The incidence of contrast-induced nephropathy (CIN) is low in patients without risk factors (< 5%) [1], but increases among patients with chronic kidney disease (CKD) [2], particularly in patients with diabetes mellitus (DM), congestive heart failure (CHF), and advanced age [3]. Additionally, the risk of developing CIN is even higher among patients with acute coronary syndrome (ACS) who undergo percutaneous coronary intervention (PCI) [4]. Incidence of CIN after a PCI is < 3% in patients without renal dysfunction but can rise to 40% in patients with chronic kidney disease besides being associated with higher in-hospital mortality [5, 6].

CIN is defined as an acute decrease in renal function after the administration of intravenous contrast without an alternative cause [7]. The most frequently used definition of CIN is based on the elevation of serum creatinine (SCr) [8]. It is important to note that SCr does not begin to rise until 50% or more of the kidney glomeruli have been affected [9]. Serum creatinine is neither sensitive nor specific because several conditions can alter its serum concentration [10, 11].

Several scoring systems have been devised to identify patients at high risk of developing CIN after a cardiac intervention [12–16]. Mehran et al created a scoring system that incorporates 8 clinical and procedural variables (hypotension, congestive heart failure, serum creatinine, DM, age > 75 years, anemia, the volume of contrast, and use of an intra-aortic balloon pump) that have been widely used and validated in different cohorts [17–20].

Given the limitations of SCr to detect patients with subclinical CIN, isolation of a biomarker for acute kidney injury after a PCI would be clinically useful. Neutrophil gelatinase associated-lipocalin (NGAL), also known as lipocalin-2 (LCN2), is a small (25-kDa) glycoprotein covalently stored in granules of mature neutrophils [21]. It is normally expressed at very low levels in a variety of human tissues, including bone marrow, uterus, prostate, salivary gland, stomach, colon, trachea, lung, liver, and kidney [22, 23]. It is rapidly released in distal tubular cells in response to inflammation or injury of nephrons [24]. It can be easily detected in the blood and urine soon after acute kidney injury [25, 26]. NGAL is emerging as a promising renal biomarker to detect patients with acute kidney injury [27]. Recent studies have shown that NGAL can be used as a diagnostic tool to detect CIN in patients that undergo an elective PCI with similar performance between urinary and serum NGAL. However, most of the studies in this group of patients used serum NGAL [25, 28–30].

However, limited data are available on the utility of NGAL to predict CIN and the need for hemodialysis in high-risk patients with ACS and this study will attempt to solve this gap in the literature. Therefore, we hypothesized that serum levels of NGAL could predict the development of CIN requiring hemodialysis after PCI in patients with ACS and high risk of developing CIN. The objective of this study is to determine whether NGAL measured 6 h post PCI can identify patients that will develop CIN and require hemodialysis. Moreover, we will assess whether NGAL levels are associated with a longer length of hospital stay and hospital mortality.

## Methods

A prospective observational study of consecutively admitted patients with ACS that underwent PCI was performed during two consecutive years at Shaio Clinic, a high-volume cardiovascular referral center in Bogota, Colombia. The local institutional ethics committee approved the study. Informed consent was obtained from all study subjects prior to enrollment. Not study procedures were performed before patient enrollment, and official inform consent was obtained.

PCI was performed using a standard protocol via either the radial or femoral artery approach by an attending interventional cardiologist. All procedures were performed using a standard dose (3–5 ml/kg) of non-ionic, low-osmolality contrast media in doses adjusted for body weight and type of cardiovascular angiogram. Patients’ interventions were performed according to local and international guidelines, not per protocol. It is essential to highlight that all patients were monitored during PCI by a certified anesthesiologist to prevent cardiovascular complications; none of the subjects included in the study developed hypotension nor cardiovascular instability during the procedure. The decision to begin hemodialysis therapy was determined by the nephrology team taking into account the following life-threatening indications for renal replacement therapy: acidosis unresponsive to medical treatment, acute severe refractory hyperkalemia, pulmonary edema and uremic complications (e.g., encephalopathy and uremic pericarditis) [31]. The nephrology team did not have access to the NGAL values during the study.

### Subjects

The study *inclusion criteria* were age > 18 years, ACS per standard definition [32], and high risk for CIN determined by a Mehran score > 11 points (Table 1).

**Table 1:** The Mehran risk score for the prediction of CIN

Mehran score periprocedural CIN risk factor	Score
Hypotension (SBP < 80 mmHg or < 1 h of inotropic support)	5
Intra-arterial balloon pump therapy	5
Chronic heart failure (NYHA III/IV or recent pulmonary edema)	5
Age < 75 years	4
diabetes mellitus	3
Anemia (male: HCT < 0.39, female: HCT < 0.36)	3
Creatinine > 1.5 mg/dL	4
OR	
Estimated glomerular filtration rate < 20 mL/min	6
Estimated glomerular filtration rate 20–40 mL/min	4
Estimated glomerular filtration rate 40–60 mL/min	2
Contrast media volume (cc)	1 point for each 100

*CIN* contrast induce nephropathy, *SBP* systolic blood pressure, *NYHA* New York heart association functional classification, *HCT* hematocrit

*Exclusion criteria* were end-stage renal disease requiring chronic hemodialysis (HD) or continuous ambulatory peritoneal dialysis (CAPD); suspected infection, sepsis or septic shock; exposure to nephrotoxic drugs or intravenous contrast medium 48 h prior to the study period; terminal disease (malignant cancer of any type or end-stage liver disease); and pregnancy.

### Definition of contrast-induced nephropathy (CIN)

CIN was defined according to criteria by the Acute Kidney Injury Network (AKIN) as an increase in SCr by ≥0.3 mg/dl (≥26.4 μmol/L) or ≥ 1.5 times baseline creatinine level within 48 h of the procedure [33]. Urine volume criteria for AKI were not applied in this study because of potential changes in urinary volume induced by diuretics in the ICU. The estimated glomerular filtration rate (eGFR) was calculated using the Modification of Diet in Renal Disease-4 (MDRD) formula [34].

### Enrollment and follow-up

After screening all patients for eligibility at the time of ICU admission, patients with high risk for CIN were treated prophylactically according to the institutional protocol for renal protection: normal saline at a rate of 1 mL/Kg/h IV; N-acetylcysteine 1200 mg IV BID on the day before and 12 h after the procedure, and sodium bicarbonate started at least 1 h before the procedure and up to 6 h after administration of contrast. All patients were followed daily until hospital discharge, and data were gathered daily using an electronic case report form for each patient.

### Clinical outcomes

Our primary aim was to determine the association of serum NGAL levels 6 h after contrast media exposure and the need for hemodialysis after PCI in patients with ACS. Our secondary outcome was to determine the association of serum NGAL levels and hospital length of stay (LOS) or hospital mortality.

### Biomarker assay

Venous blood samples measured serum creatinine (SCr) and blood urea nitrogen (BUN) every 24 h after ICU admission. Blood was drawn prior to PCI and 6, 24, and 48 h after the procedure to determine levels of SCr, BUN, and NGAL. Serum NGAL was measured using a commercially available kit (Alere™ Triage® NGAL immunoassay), immediately after blood sample collection according to the manufacturer’s instructions.

### Statistical analysis

We used Fisher’s exact test to compare categorical variables and the non-parametric test (Man-Whitney U Test) to evaluate continuous variables. Values are expressed as medians (IQR). Statistical significance was defined as a *p*-value ≤0.05. A receiver operating characteristic (ROC) curve was developed to assess the accuracy of NGAL levels to -index to identify the best cut-off at 6 h post PCI of NGAL. All statistical analyses were performed with IBM SPSS, Statistics for Mac, version 22.0. Armonk, NY: IBM Corp.

## Results

**Fig. 1:**
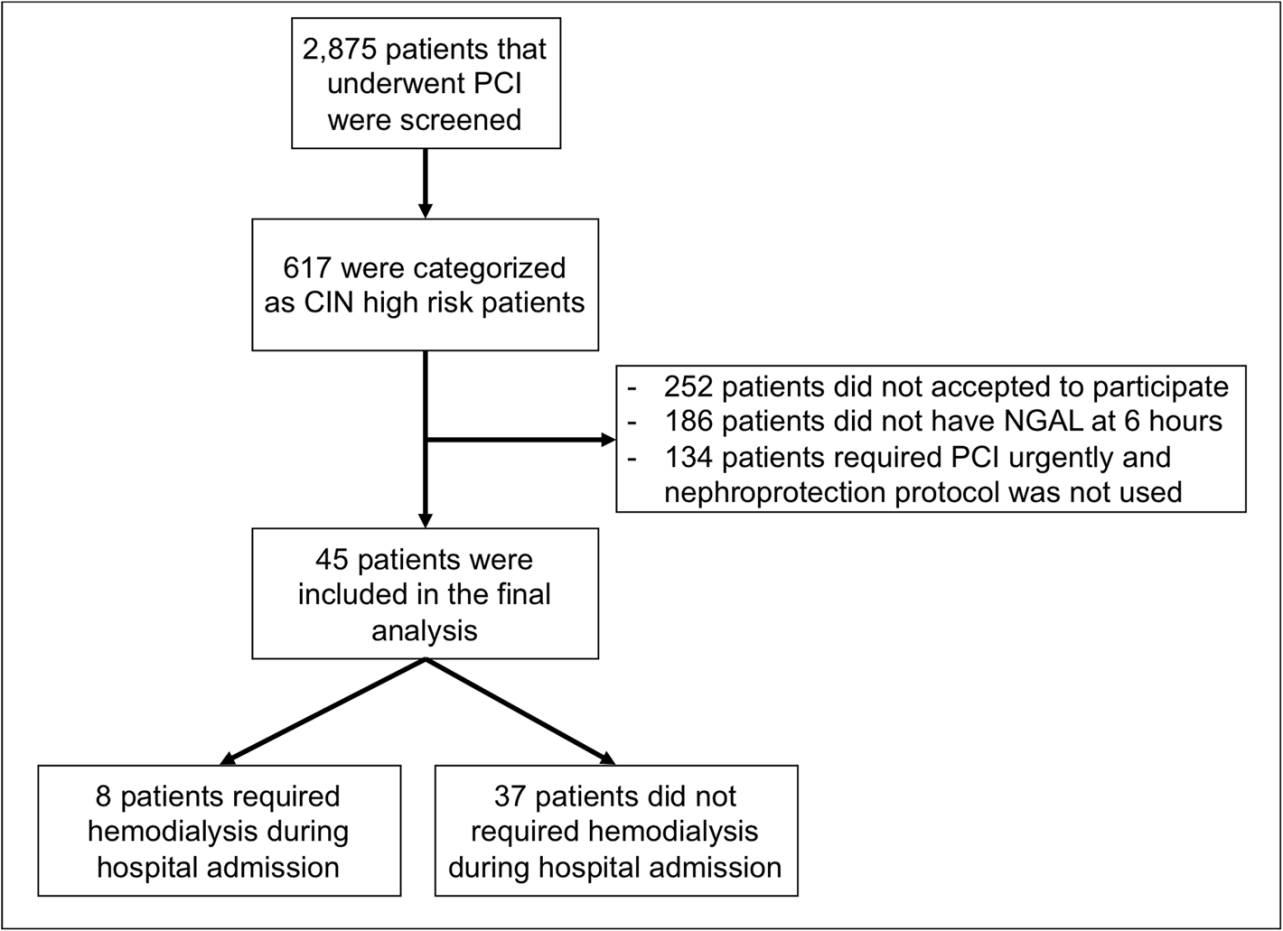
Study flow chart. Flow diagram of the patients with the acute coronary syndrome (ACS) that underwent PCI that entered the study

**Fig. 2:**
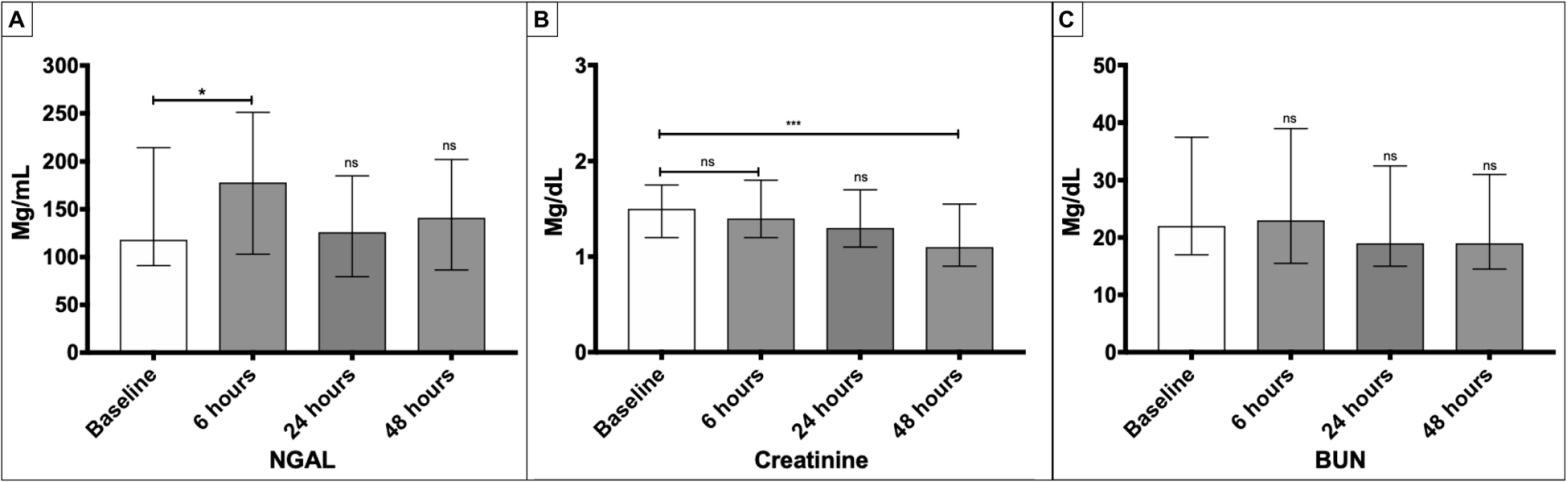
Serial measurements of NGAL, creatinine, and BUN at baseline, 6, 24, and 48 h. Box plots of serum NGAL, creatinine, and BUN at baseline, 6 h, 24 h and 48 h. **a**. NGAL levels started to rise at 6 h and then decreased at 24 and 48 h. **b**. Creatinine values did not rise at any interval. **c**. BUN levels also did not rise at any time interval. NS: Not significant; P: < 0.05

A total of 2875 PCIs were performed during the study period. We identified 617 patients as potential study subjects; however, only 45 patients met the inclusion/exclusion criteria and were enrolled as study subjects (Fig. 1). A majority of the subjects were men (60%) and older than 70 years old. During the median 12.2 days of follow-up of all study subjects, 8 patients required hemodialysis therapy, and 3 patients died. None of the patients develop cardiogenic shock nor required cardiac surgery. The baseline characteristics of study subjects are presented in Table 2, and the serial measurements of NGAL, creatinine, and BUN at baseline, 6, 24, and 48 h are shown in Fig. 2.

**Table 2:** Baseline characteristics of patients with acute coronary syndrome (ACS) stratified according to the requirement of hemodialysis during hospital admission

Characteristic	No Hemodialysis (***n*** = 37)	Hemodialysis (***n*** = 8)	***p*** Value
**Demographic**			
Male	19 (51)	8 (100)	**0.01**
Age, median (IQR)	76 (69, 80)	71 (63, 79)	0.41
**Comorbid conditions*,*n (%)**			
Obesity	7 (19)	1 (12)	0.66
Hypertension	30 (81)	7 (87)	0.66
Active cancer	2 (5)	0 (0)	0.31
Atrial fibrillation	4 (10)	2 (22)	0.28
Chronic heart failure	15 (40)	6 (75)	0.07
COPD	4 (10)	0 (0)	0.37
Chronic kidney disease	3 (8)	1 (12)	0.30
Diabetes mellitus	16 (43)	5 (62)	0.32
Hyperlipidemia	27 (73)	6 (75)	0.90
Hypothyroidism	10 (27)	3 (37)	0.55
Tobacco use	12 (32)	4 (50)	0.34
At admission, median (IQR*)			
Left ventricular ejection fraction	45 (21, 55)	32 (30, 53)	0.89
MDRD4	46 (40, 47)	36 (31, 46)	0.14
Creatinine	1.15 (1.2, 1.6)	1.75 (1.2, 2.1)	0.27
BUN	21 (15, 35)	37 (19, 57)	0.09
Hemoglobin	14.10 (12.6, 15.9)	12.55 (11.5, 14.1)	0.09
Platelets	219 (193, 282)	261 (200, 281)	0.63
**Admission diagnosis, n (%)**			
NSTEMI	24 (65)	7 (87)	0.21
STEMI	3 (8)	1 (12)	0.69
Unstable angina	10 (27)	0 (0)	0.95
**Clinical outcomes, n (%)**			
Discharge hemodialysis	0 (0)	1 (12)	**0.03**
In-hospital mortality	0 (0)	3 (37)	**< 0.01**

*NSTEMI* non-ST-elevation myocardial infarction, *STEMI* ST-segment elevation myocardial infarction, *IQR* interquartile ratio, *COPD* chronic obstructive lung disease; *BUN* blood urea nitrogen, *MDRD4*, 4-variable modification of diet in renal disease study group formula

Among the patients that required versus did not require hemodialysis, there were no differences in hemoglobin levels or platelets counts. However, subjects in the hemodialysis group had higher albumin levels, lower glomerular filtration rates at admission determined per MDRD-4 formula, and lower left ventricular ejection fraction. These subjects also had a longer hospital LOS, and one subject continued to requiring hemodialysis at hospital discharge. Finally, among subjects that required hemodialysis, 3 died. Serum concentrations of NGAL at 6 h were higher in patients whom died during hospital admission (341.0 mg/mL [311–350] vs. 171.5 mg/mL [100–230], *p =* 0.007). However, serum concentrations of NGAL were not different in patients with CIN who died (340.0 mg/ml [220–392] vs. 341.0 mg/mL [311–341], *p =* 0.1). As expected, patients that required hemodialysis had a longer hospital LOS (17 days [+/− 5] vs. 10 days [+/− 7], *p =* 0.008).

**Fig. 3:**
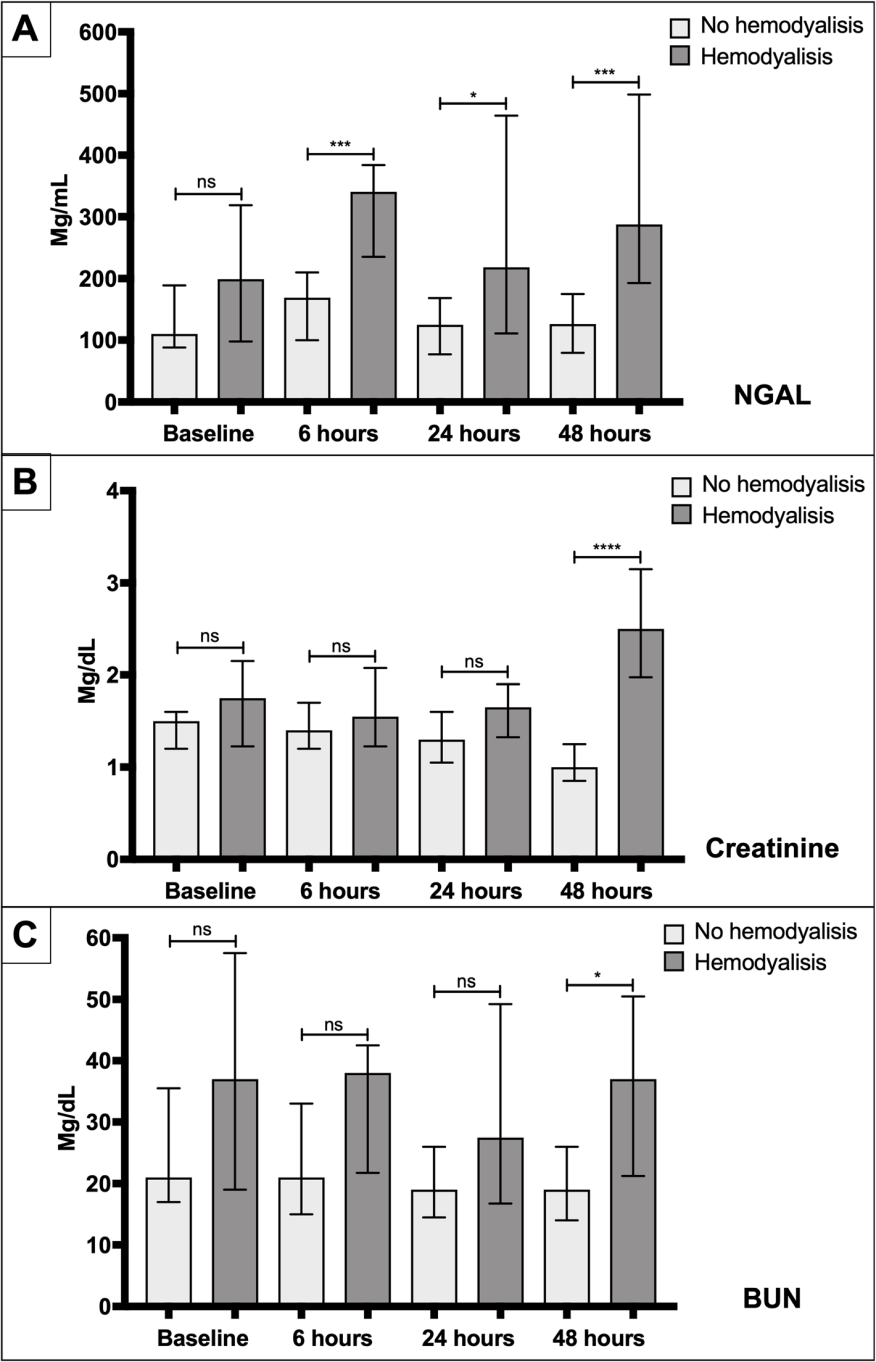
Serial measurements of NGAL, creatinine, and BUN at baseline, 6, 24, and 48 h in hemodialysis patients versus those who did not require hemodialysis. Box plots of serum NGAL, creatinine and BUN at baseline, 6 h, 24 h, and 48 h in the patients who required hemodialysis and those who did not. **a**. NGAL levels started to rise at 6 h with higher values in those patients who require hemodialysis. **b**. Creatinine values only started to rise at 48 h in those patients who require hemodialysis. **c**. BUN levels did not show a rising pattern in either group. NS: Not significant; *p*: < 0.05

Regarding serum biomarker levels at 6 h, the median (IQR) for serum concentration of NGAL was significantly higher in subjects that required hemodialysis versus those that did not require hemodialysis (340.5 mg/mL [235–384] vs. 169 mg/mL [100–210], *p =* 0.001) (Fig. 3). BUN was also higher in the hemodialysis group versus those subjects not requiring hemodialysis, but the difference was not statistically significant. The median serum concentration of creatinine at 6 h was similar between subjects that required hemodialysis versus those that did not require hemodialysis (1.55 [1.22–2.07] vs. 1.40 [1.20–1.70], *p* = 0.37). These data demonstrated that elevated serum levels of NGAL at 6 h predicted the need for hemodialysis with an area under the curve (AUC) of 0.858. Using the Youden index, we identified that the best cut-off at 6 h post PCI of NGAL is 281 mg/dL. With this cut-off, the sensitivity and specificity were 0.75 and 0.95, respectively, to predict the requirement for hemodialysis.

## Discussion

To the best of our knowledge, this is the first study available in medical literature to assess the role of serum NGAL as an early biomarker of CIN after PCI in high-risk patients. The main finding of our study is that serum levels of creatinine and BUN, traditional biomarkers of renal injury, could not detect early development of CIN. In contrast, serum concentrations of NGAL at 6 h post-PCI could detect patients that are at high risk of developing CIN requiring hemodialysis during hospitalization. Moreover, we also found that NGAL levels were higher in patients who developed CIN and died during hospital admission. This finding is clinically relevant because using serum NGAL concentration, physicians could identify patients at risk of developing CIN that will require hemodialysis early and may benefit from prompt renal specialist consultation and medical interventions, such as optimization of intravascular volume status and avoidance of nephrotoxic medications.

Although our study is the first to use serum NGAL for early detection of CIN in high-risk patients with ACS undergoing PCI; our findings build on prior knowledge that has shown that NGAL is superior to serum creatinine or BUN for early diagnosis of acute kidney injury and CIN. A recent meta-analysis showed that NGAL had an excellent predictive utility for CIN with AUCs of 0.91 for serum NGAL and 0.94 for urinary NGAL [35]. However, in this meta-analysis, authors only included 4 studies that used serum NGAL in adults, and all 4 studies had small sample sizes [35]. Additionally, these studies were in patients who underwent elective PCI, and only one study used the same definition of CIN as our study. Besides this, regarding renal protection protocols, in two of them, patients received normal intravenous saline at a rate of 1 mL/kg per hour previous to the procedure and in only one study patients also received oral N-acetylcysteine (NAC) 600 mg twice daily for 3 days [28, 30, 36]. A more recent study by Nguyen et al. with unselected patients with ST-elevation myocardial infarction treated by PCI and were all patient receive only 1000 mL of physiological saline given at a rate of 0.6 mL/kg per hour for 24 h except in those patients in Killip class III or IV; plasmatic NGAL did not provide additional value regarding CIN prediction compared with other risk [37].

In contrast, all of our study subjects received per-protocol treatment for renal protection to prevent CIN that included normal saline at a rate of 1 ml/Kg/h IV; N-acetylcysteine 1200 mg IV BID 24 h before and 12 h after the procedure, and sodium bicarbonate started at least 1-h pre-procedure and continued up to 6 h post-procedure. Moreover, our study included only high-risk patients identified by Mehran score > 11 points, whereas other studies included lower-risk patients, which constitutes the strength and novelty of our results.

The importance of early detection of CIN is that several observational studies have demonstrated that in-hospital mortality is five times higher in patients with CIN that patients who do not have CIN. Added to the above, observational studies have shown that as many as 20% of patients who develop CIN suffer a persistent worsening of renal function [5, 38, 39]. Therefore, early detection of CIN could mean an early consultation to a renal specialist because it has been demonstrated that a delay in nephrology consultation contributes to higher mortality in acute kidney injury [40].

Several urinary and serum biomarkers have been proposed to identify patients at risk of dying due to CIN. However, the results are controversial and not conclusive. In our study, we found that patients with higher concentrations of serum NGAL had higher mortality. Even though this is an exciting finding, our study was not powered nor designed to predict mortality; thus, this finding should be interpreted with caution. However, this might open the possibility to design more prominent, multicentric, prospective studies to evaluate whether NGAL may be used as a prognosis biomarker in patients with CIN and, more specifically, in patients with AKI due to CIN.

Our study has important strengths and limitations that need to be recognized. One of the strengths of our study is that the setting was a highly specialized cardiovascular hospital with established protocols to identify and intervene on patients at risk of CIN. Another strength of our study is the inclusion of only high-risk patients, an important group of patients frequently excluded from other studies. However, it is essential to recognize that the small sample size limits our study due to the recruitment of a specific high-risk patient population. This also limits our ability to the performance of all statistical tests to assess the performance of NGAL as a diagnostic marker in the early detection of patients with CIN requiring hemodialysis. Moreover, only three patients died in our study; thus, we cannot conclude, nor hypothesize the role of NGAL in this regard. It is important to mention that NGAL has been recently associated with heart failure and coronary artery disease possible as a manifestation of inflammation [41]; and essential aspect that needs to be kept in mind because this condition could develop a false positive scenario. Besides this, some patients that required hemodialysis had higher baseline sCr, which could imply some undiagnosed cases of the chronic kidney, which could have an impact on the initial value of NGAL. Nevertheless, the discriminatory power of NGAL at 6 h superior to creatinine levels at 6 h to predict the need for hemodialysis. *Acute kidney injury is a widely used and accepted definition. However, there are several definitions, and there is controversy about which is the most representative classification. Thus, we did not asses AKI in patients that did not require hemodialysis. Readers should be aware that the data presented in this study only represent patients with severe CIN requiring hemodialysis, and these data should not be extrapolated to other patients.* Finally, *not all hospitals can ensure nephroprotection in high-risk patients within the first 6 h of hospital admission. Therefore, the results presented in this study might not be generalizable for all hospitals*.

## Conclusion

In summary, our study has demonstrated that in patients with ACS undergoing PCI that are at high risk of developing CIN, an elevated serum level of NGAL 6 h after contrast exposure predicts the development of acute renal failure requiring hemodialysis. Early detection of acute kidney injury may prompt clinicians to seek early renal consultation and initiate aggressive therapies to reduce the risk of progression of renal failure. Additional studies are needed to confirm our findings and identify potential therapeutic interventions that may delay the progression of CIN in high-risk patients.

## Data Availability

Not applicable.

## Abbreviations

ICU: Intensive care unit
NGAL: Neutrophil Gelatinase Associated Lipocalin
CIN: Contrast-induced nephropathy
ACS: Acute coronary syndrome
PCI: Percutaneous coronary intervention
CKD: Kidney disease
DM: Diabetes mellitus
CHF: Congestive heart failure
SCr: Serum creatinine
HD: Hemodialysis
CAPD: Continuous ambulatory peritoneal dialysis
eGFR: Estimated glomerular filtration rate
MDRD: Modification of diet in renal disease-4
LOS: Length of stay
